# Work-related correlates of occupational sitting in a diverse sample of employees in Midwest metropolitan cities

**DOI:** 10.1016/j.pmedr.2017.03.008

**Published:** 2017-03-22

**Authors:** Lin Yang, J. Aaron Hipp, Jung Ae Lee, Rachel G. Tabak, Elizabeth A. Dodson, Christine M. Marx, Ross C. Brownson

**Affiliations:** a Division of Public Health Sciences, Department of Surgery, Washington University School of Medicine, USA; b Department of Epidemiology, Center for Public Health, Medical University of Vienna, Austria,; c Department of Parks, Recreation, and Tourism Management, College of Natural Resources, North Carolina State University, USA; d Brown School, Washington University in St. Louis, USA; e Prevention Research Center in St. Louis, Washington University in St. Louis, USA; f Agricultural Statistics Laboratory, University of Arkansas, USA; g Alvin J. Siteman Cancer Center, Washington University School of Medicine, Washington University in St. Louis, USA; h Center for Geospatial Analytics, North Carolina State University, USA

**Keywords:** Worksite support and policies, Occupational sitting, Occupation, Physical activity

## Abstract

The worksite serves as an ideal setting to reduce sedentary time. Yet little research has focused on occupational sitting, and few have considered factors beyond the personal or socio-demographic level. The current study i) examined variation in occupational sitting across different occupations, ii) explored whether worksite level factors (e.g., employer size, worksite supports and policies) may be associated with occupational sitting.

Between 2012 and 2013, participants residing in four Missouri metropolitan areas were interviewed via telephone and provided information on socio-demographic characteristics, schedule flexibility, occupation, work related factors, and worksite supports and policies. Occupational sitting was self-reported (daily minutes spent sitting at work), and dichotomized. Occupation-stratified analyses were conducted to identify correlates of occupational sitting using multiple logistic regressions.

A total of 1668 participants provided completed data. Those employed in business and office/administrative support spent more daily occupational sitting time (median 330 min) compared to service and blue collar employees (median 30 min). Few worksite supports and policies were sitting specific, yet factors such as having a full-time job, larger employer size, schedule flexibility, and stair prompt signage were associated with occupational sitting. For example, larger employer size was associated with higher occupational sitting in health care, education/professional, and service occupations.

Work-related factors, worksite supports and policies are associated with occupational sitting. The pattern of association varies among different occupation groups. This exploratory work adds to the body of research on worksite level correlates of occupational sitting. This may provide information on priority venues for targeting highly sedentary occupation groups.

## Highlights

• Work-related factor and worksite policies related to occupational sitting.
• Daily occupational sitting time was the highest among business and office workers.
• Employer size, flexibility, and stair prompt signage were associated with sitting.
• Worksite support and policies are in need to reduce sedentary behavior sustainably.

## 1 Introduction

Sedentary behaviors are linked to adverse health outcomes such as chronic disease risk factors (Helmerhorst et al., 2009, Jakes et al., 2003, Sisson et al., 2009, Thorp et al., 2010, Wijndaele et al., 2009, Wijndaele et al., 2010b), the development of chronic diseases (Beunza et al., 2007, Hu et al., 2001, Hu et al., 2003), and mortality (Dunstan et al., 2010, Katzmarzyk et al., 2009, Wijndaele et al., 2010a), possibly independent from levels of physical activity (Healy et al., 2008, Helmerhorst et al., 2009, Hu et al., 2003, Jakes et al., 2003, Katzmarzyk, 2010). Sedentary behavior is distinct from physical inactivity. For example, prolonged sitting (i.e., occupational sitting, watching TV) may exist among people who are physically active by engaging in sufficient recreational activity. Therefore, reducing prolonged sitting time and interrupting sitting time by active breaks is recommended even for adults who meet the recommended level of physical activity (Department of Health, 2011, Garber et al., 2011).

Historically, epidemiologic studies examined physical demands at work and leisure-time activity in relation to the rate of developing outcomes such as coronary heart disease and all-cause mortality (Fox and Skinner, 1964, Hartley and Llewellyn, 1939, Morris et al., 1973, Morris et al., 1953). Nevertheless, the majority of current studies on sedentary behavior have focused on sitting during leisure time, e.g., TV viewing, rather than occupational sitting (Dunstan et al., 2010, Hu et al., 2003, Jakes et al., 2003). Available evidence links sitting at work to obesity (Hu et al., 2003, Mummery et al., 2005) and diabetes (Hu et al., 2003). Workplaces may be an ideal setting to reduce sitting time through implementing worksite policies or improving the work environment infrastructure, given that working adults may spend 8 h or more per day at work during working days (Carnethon et al., 2009, van Uffelen et al., 2010). Along with industrialization and the development of modern technology, many adults are employed in occupations that mainly involve sitting, particularly in developed countries (Owen et al., 2010). In addition, the ecological model has identified environmental and policy approaches as the most promising strategies to influence physical activity behavior at the population level (Sallis et al., 1998, Sallis et al., 2006). Therefore, there is a need to investigate the potential to reduce sitting time through workplace environment and policy support.

A recent review (Smith et al., 2016), which included 41 studies, examined the correlates of occupational physical activity and sedentary behavior, in order to synthesize current evidence and inform intervention design specific to workplace-based settings. The review is timely because it reveals a critical research gap, which is the lack of studies focusing on occupational sedentary time, namely occupational sitting. More importantly, among six studies that included occupational sitting, only one investigated factors beyond the personal or socio-demographic level (Tissot et al., 2005). Further, the time of data collection of that study was 1998, which is almost 20 years ago, thus updated data and research on this topic are necessary.

Physical demands and sedentary needs and behaviors vary by occupation; however previous studies have often overlooked occupational differences in sitting time. Thus, in the current study, we aim to examine the variation in occupational sitting across different occupations, and further investigate work-related factors in relation to occupational sitting across different occupations. We also explore whether specific worksite supports and policies for active workplaces may influence occupational sitting in a large sample of adults in Missouri metropolitan areas.

## 2 Method

### 2.1 Study population and study design

The participants in this study were from the Supports at Home and Work for Maintaining Energy Balance (SHOW-ME) study, a cross-sectional study designed to understand the environmental, programmatic, and worksite policy influences on employees' obesity status. The study design has been described in detail elsewhere (Yang et al., 2014). In brief, between 2012 and 2013, 2015 participants employed and living in four Missouri metropolitan areas (St. Louis, Kansas City, City of Springfield, and City of Columbia) were recruited using list-assisted telephone random-digit-dialing methods. The first eligible adult who volunteered to participate was sampled in each household. The eligibility criteria included: aged 21–65 years; employed outside of the home at one primary location, employed for 20 or more hours per week at one site with at least 5 employees; not pregnant; and had no physical limitation that prevented walking or bicycling in the previous week. Recruited participants completed a survey over the phone which was developed for the SHOW-ME study and based on existing self-reported and environmental assessment instruments, and input from a Questionnaire Advisory Panel (QAP) comprised of experts in survey development, nutrition/food environment, physical activity, transportation, and worksite environmental intervention (Hoehner et al., 2013). The study design was approved by the institutional review boards of Washington University in St. Louis and University of Missouri-Columbia. All participants provided informed consent.

## 3 Measures

Participants self-reported their occupation, as well as job-related features, such as whether they supervised others or had a flexible work schedule. Research team members coded these occupations using the US Bureau of Labor Statistics' Standard Occupational Classification (SOC), and referencing the O*NET OnLine resource for detailed descriptions of each occupation. Based on the SOC codes, SOC *Job Families* (where categorization is based upon similar work performed as well as similar required education and skills), and team consensus, the research team combined occupation codes into six broad occupation categories: healthcare, business, education/professional, service, blue collar, office/administrative support.

## 4 Main outcome/dependent variable

### 4.1 Occupational sitting

The telephone survey incorporated questions adapted from the Australian Longitudinal Study on Women's Health (Marshall et al., 2010), which records the frequency and duration of sedentary activities at work, at home, and during travel to/from work. Time spent sitting at work was determined by the following question: “Please estimate how many hours you spent sitting each day while at work.” Time spent sitting at work was recorded in hours and minutes, and then recoded to total minutes per day. Due to its non-linearity, data were tested via scatter plots, box plots, frequency tables, and a square root transformation procedure on the occupational sitting variable. In each occupation category, we dichotomized daily occupational sitting time to sitting less and sitting more approximating the median cut-off value to indicate the different levels of sedentary behavior involved at work for each participant. Median cut off score was used because it appears to be the appropriate measure of central tendency given the distribution of the outcomes variable. In the occupation group stratified analyses, we used median cut off scores of occupational sitting in each occupation group. By doing so, we attempted to account for the variation of occupational sitting due to different occupations. Due to the nature of different occupational categories, the median cut-off values vary from 30 min to 330 min per week to ensure a balanced sample size between the two binary responses for reliable estimation. The median cut-off value for the overall sample was 180 min per week.

## 5 Exposures/correlates/independent variables

### 5.1 Work related factors

Information on household income, employer size, and whether they were working full time was self-reported by participants. Household income was collapsed into three groups approximating tertiles, which were: less than $39 k, between $40 k and $74 k, and more than $75 k. Employer size categories were also collapsed into four groups approximating quartiles, which were: between five and 49 employees, between 50 and 200 employees, between 201 and 499 employees, and > 500. Participants also reported their schedule flexibility at work which was dichotomized into yes and no. Work-related physical activity was assessed using selected questions from the International Physical Activity Questionnaire (IPAQ). IPAQ has been tested internationally for reliability (Spearman's ρ ~ 0.8) and validated with objective measures (median ρ ~ 0.3); these values are comparable to values found in other validation studies of self-reported data (Craig et al., 2003). Work-related physical activity refers to activities completed as part of paid or unpaid work, namely physical demands of work activities. We dichotomized weekly minutes spent in work-related physical activity into < 150 min per week (insufficiently active) or 150 or more minutes per week (sufficiently active), to determine whether participants were active at work, independent of sedentary time (CDC, 2011).

### 5.2 Worksite supports and policies

Worksite supports and policies were determined using 18 questions asking whether specific policy, programmatic, and environmental supports for physical activity were available at the worksite. These questions have been used elsewhere and shown moderate to almost perfect reliability in measuring worksite policies, programs, and environments supporting physical activity (Hoehner et al., 2013). We selected three questions that were associated with the level of physical activity engagement in the study sample (under review) to include in the current analysis: did the worksite offer physical activity breaks during the work day, were there stair prompts present at the worksite, and were maps of the workplace neighborhood available.

## 6 Covariates

### 6.1 Individual socio-demographic variables

Participants self-reported age, gender, race and ethnicity, weight, and height. Age was categorized into 21–44, 45–54, and 55–65 years old, respectively, approximating the tertile cut-off values. Body mass index (BMI) was calculated as weight/height^2^ (kg/m^2^) and was categorized to under or normal weight (BMI < 25.0), overweight (25.0 ≥ BMI > 30.0) and obese (BMI ≥ 30.0) (World Health Organization, 2000)

## 7 Analysis

We summarized participant characteristics, sitting time spent at work and work related factors using frequencies and percentages. We stratified the analyses by occupation group to account for different work-related factors. The associations between correlates and occupational sitting were estimated using logistic regression. Due to reduced sample size after stratification, all ordinal correlates were treated as continuous variables when included as control variables to ensure statistical power. Sensitivity analysis was carried out to compare all explanatory variables as categorical and continuous and both derived similar conclusions on the direction and significance of the associations. Thus we reported the continuous measures for the model for simplicity with a smaller number of parameters. All analyses were adjusted for individual socio-demographic variables. The statistical significance level was set to 0.05, and therefore 95% confidence intervals are reported. All analyses were performed using R version 3.0.3 (http://www.R-project.org/, Vienna, Austria).

## 8 Results

In total, 1668 participants completed data on self-reported sitting time spent at work, individual characteristics, work-related factors, worksite supports and policies, and type of occupation and industry. The characteristics of the participants are shown in Table 1. There were more female (66.6%) than male participants, and the majority of the sample was white (63.7%) and overweight (32.2%) or obese (34.1%).

Table 2 shows the adjusted odds ratios and confidence intervals for six logistic regression models by occupation group: health care, business, education/professional, service, blue collar, office/administrative. Employees in different occupation groups spent different lengths of time in occupational sitting, with the median daily sitting time higher in business and office/administrative support (330 min) and lower in the others, with service and blue collar (30 min) reporting the lowest of all.

After adjusting for age, race, BMI, and household income, our analyses showed that having a full time job was positively associated with a higher level of occupational sitting in all occupations except for health care (aOR: 1.80, 95% CI: 0.93–3.50). Bigger employer size appears to be significantly associated with a higher level of occupational sitting in two occupation groups: education/professional (aOR: 1.66, 95% CI: 1.26–2.21), and blue collar (aOR: 1.70, 95% CI: 1.24–2.37). Except for the null finding among blue collar employees, all other occupations reported lower levels of occupational sitting related to higher levels of physical activity involved at work.

Among the worksite supports and policies that were associated with work-related physical activity, two appeared to be associated with occupational sitting. Worksites offering flexible time for physical activity during the day was associated with higher levels of occupational sitting in education/professional (aOR: 1.76, 95% CI: 1.40–2.23) and blue collar (aOR: 1.27, 95% CI: 1.02–1.59) occupations. Worksites offering signs to encourage stair use showed a higher level of occupational sitting in education/professional (aOR: 2.28, 95% CI: 1.07–5.00), service (aOR: 2.66, 95% CI: 1.20–6.14), and office/administrative support (aOR: 2.29, 95% CI: 1.02–5.29) occupations.

Results from sensitivity analyses modeling correlates as categorical variables confirmed previous findings. Interaction analyses revealed only one significant interaction between individual characteristics and significant work-related factors among all occupations. In the office/administrative support occupations, having a full time job was associated with a higher level of occupational sitting, yet this association differs between men and women with a stronger association in women.

## 9 Discussion

We examined the occupational sitting pattern among six different occupation groups: health care, business, education/professional, service, blue collar, office/administrative support. We also explored how these patterns are associated with work-related factors, and worksite supports and policies. Employees in different occupation groups spent different lengths of time in occupational sitting, which varied between 30 min to 330 min per day. After adjusting for age, race, BMI and household income, our analyses showed that a having a full time job was positively associated with a higher level of occupational sitting in all occupations except for health care. Other important correlates of a higher level of occupational sitting were bigger employer size and a lower level of physically demanding work. Worksite support and policies, such as offering scheduling flexibility and signs to encourage stair use showed higher levels of occupational sitting in at least two occupational groups.

The proportion of obese participants (34.1%) in the current sample is similar to the proportion (36.9%) in a recent national study (Yang and Colditz, 2015). Self-reported occupational sitting duration differs by types of occupation. Previous studies reported 140 min/day sedentary time spent at work across various occupations and business sectors in a Dutch population (Jans et al., 2007), and 220 min/day in Belgium (De Cocker et al., 2014). The Belgium study also found a higher level of occupational sitting in white-collar/professional occupation; this is consistent with our findings, which showed a higher level of occupational sitting in business, education/professional, and office/administrative support occupations. A limited number of studies reported correlates of occupational sitting, yet most included only intrapersonal or socio-demographic factors, such as age, education level, income, and smoking (Smith et al., 2016). Few studies reported on work-related correlates of occupational sitting. Our findings are consistent with a study conducted in German men and women (Wallmann-Sperlich et al., 2014), that reported an inverse association between work-related physical activity and work-related sitting time. This association was seen in all occupations in our study, except blue collar. Indeed, compared to other included occupations (health care, business, education/professional, service, and office/administrative support), blue collar occupations involve a much higher level of physical demand that limits the variation of work-related physical activity, reducing the ability to detect an observed association with sitting time.

There is a dearth evidence on the association between worksite supports and policies and occupational sitting. However such worksite supports and policies have the potential to impact working adults' daily behavior. The strongest (adjusted OR > 2.0) finding on worksite supports and policies is the positive association between stair prompt signage and occupational sitting across education/professional, service and office/administrative support occupations. The reported association indicated that the appearance of the stair prompt signage is associated with a 2-fold or higher likelihood of having more occupational sitting time. The point-of-decision prompt, stair prompt signage in this case, has been studied over the last several decades and was recommended to increase physical activity in order to deter sedentary behavior (Russell et al., 1999). A recent systematic review suggested that stair-prompts increased stair use in 64% of studies conducted in worksite settings. Specifically, combining motivational and directional signs in worksites showed increased stair use in 83% of reviewed studies (Bellicha et al., 2015). Given that our data are cross-sectional, it is not possible to determine the causality of the observed association. The observed association between the presence of stair prompt and longer occupational sitting time could be due to several reasons. It is possible that worksites with larger number of employees are more likely to adopt and proactively promote stair prompts as a worksite support and policy, and these large worksites are more likely to be office jobs that require more sitting. However we were not able to access this questions stratifying by the number of employees within each occupation due to insufficient sample size. Alternatively, it is possible that employees may over compensate time spent in sitting at work if they regularly take the stairs. Nevertheless, stair prompts are commonly used as a strategy to promote physical activity, not target at sedentary behavior. In fact, current worksite supports and policies mostly target physical activity; few are sedentary behavior/sitting specific.

To our knowledge, this is one of the first studies investigating the correlates of occupational sitting including a range of variables from work-related and worksite supports and policies factors, adjusting for individual factors. We stratified our analysis by occupation group attaining a considerable sample size in each group in our models, which provides evidence to support developing further research and interventions targeting employees in certain occupations for dissemination. Owing to the cross-sectional design, the findings from this study lack the ability to determine the causal relationship of observed associations. The measurement instruments used in our study are reliable, although the data used in this analysis depends on self-reported sitting time at work, which may be subject to response bias (Marshall et al., 2010). Finally, due to the sampling strategy and restriction to certain geographic areas, the generalizability of the findings may be limited to Missouri metropolitan areas.

Our findings provide evidence to support developing further research and interventions targeting employees in certain occupations for dissemination. For example, individuals in business, education/professional, and office/administrative support occupations spent more time sitting at work compared to health care, service, and blue collar employees. Therefore, the worksite supports and policies need to be tailored to different occupations. Stair prompt signage was strongly associated with higher sitting time in three occupations. Despite previous research that reported the effectiveness of such point-of-decision intervention in promoting physical activity through stair use, our data suggested that having the stair prompt signage was associated with higher levels of occupational sitting. It is possible that stair promote signage is an indicator of large employer size. Small worksites with none or one floor might not have the stair prompt signage. Future studies should include larger sample of employees in highly sedentary occupations, to explore if this association could be explained by the proactive promoting of stair prompts in large organizations. Furthermore, studies need to include more work-related characteristics to understand the constraints of different occupations. That is to say, if the nature of the work does not allow insertions of breaks from sedentary behavior, it is unlikely worksite supports and policies can be effective in reducing sedentary behavior. In addition, studies to evaluate worksite interventions to reduce sedentary behavior should incorporate measures of standing time, stepping, and light-intensity physical activity, which might be replaced by reduced sitting time rather than moderate-to-vigorous physical activity. Such measures are currently being explored and tested in studies of small sample size (Sanders et al., 2016, Spinney et al., 2015). Using longitudinal designs, future studies should explore the potential of interventions to reduce occupational sitting targeting high risk occupations (business, education/professional, and office/administrative support), including supports and policies that can be initiated and implemented through organizational efforts. These studies are ideally incorporated with psychological factors, improved measurement of sedentary behavior including breaks that interrupt sedentary time, and work-related factors to understand the effectiveness of delivering worksite support and policy interventions.

## 10 Conclusion

Work-related factors and worksite supports and policies are associated with occupational sitting. The pattern of association varies among different occupation groups. This exploratory work adds to the body of evidence on the worksite level correlates of occupational sitting, and may provide venues to reduce sedentary behavior through worksite intervention, targeting highly sedentary occupation groups.

## Conflict of interests

The authors declare there is no conflict of interest.

## Figures and Tables

**Table 1:** Descriptive of socio-demographic characteristics, work-related factors and workplace supports and policies of participants by occupational sitting category, Missouri, US, 2011–2012.

	Occupational sitting time				Total	Chi-squared test
	≤ 180 min		≤ 180 min			
	n	%	n	%		*p*-Value
*Age*						
21–44 years	393	56.8	299	43.2	692	0.028
45–54 years	333	51.1	319	48.9	652	
55–65 years	312	50.0	312	50.0	624	

*Sex*						
Male	688	51.1	658	48.9	1346	0.074
Female	358	55.5	287	44.5	645	

*Race*						
Other	454	61.9	279	38.1	733	< 0.001
White	581	46.9	657	53.1	1238	

*BMI*						
Not Obese (< 30)	671	53.5	583	46.5	1254	0.145
Obese (≥ 30)	316	49.8	318	50.2	634	

*Employment*						
Part time	377	69.3	167	30.7	544	< 0.001
Full time	670	46.2	779	53.8	1449	

*Employer size*						
5–49	366	59.8	246	40.2	612	< 0.001
50–200	361	59.7	244	40.3	605	
200–499	113	43.3	148	56.7	261	
500 and up	153	36.1	271	63.9	424	

*Household income*						
$0 k–$39 k	294	76.2	92	23.8	386	< 0.001
$40 k–$74 k	425	53.1	376	46.9	801	
$75 k–up	269	39.5	412	60.5	681	

*Work-related physical activity*						
Inactive	288	29.6	685	70.4	973	< 0.001
Active	759	74.4	261	25.6	1020	

*Schedule flexibility at work*						
No	331	62.8	196	37.2	527	< 0.001
Little	187	45.8	221	54.2	408	
Some	236	48.5	251	51.5	487	
A lot	173	46.5	199	53.5	372	
Completely	117	60.0	78	40.0	195	

*Physical activity break*						
No	854	51.4	809	48.6	1663	0.007
Yes	177	60.0	118	40.0	295	

*Stair prompt signage*						
No	798	52.2	732	47.8	1530	0.754
Yes	227	53.2	200	46.8	427	

*Maps of workplace neighborhood*						
No	839	53.3	735	46.7	1574	0.239
Yes	182	49.7	184	50.3	366	

**Table 2:** Adjusted odds ratios in multiple linear regression analyses of the association between occupational sitting and socio-demographic characteristics, work-related factors, and worksite supports and policies, in six occupation groups, Missouri, US, 2011–2012 (N = 1597).

Occupational sitting reference group (daily minutes)^1^	Health care		Business		Education/professional		Service		Blue collar		Office/admin support	
	n = 222		n = 277		n = 304		n = 281		n = 232		n = 281	
	≤ 90 min (n = 104)		≤ 330 min (n = 121)		≤ 210 min (n = 157)		≤ 30 min (n = 139)		≤ 30 min (n = 98)		≤ 330 min (n = 127)	
	aOR	95% CI	aOR	95% CI	aOR	95% CI	aOR	95% CI	aOR	95% CI	aOR	95% CI
*Work-related factors*												
Full time (ref: no)												
Yes	1.80 (0.93–3.50)		3.00 (1.33–6.96)⁎⁎		1.73 (0.84–3.50)		3.94 (2.26–6.99)⁎⁎		1.57 (0.78–3.17)		3.69 (1.77–7.92)⁎⁎	
Employer Size (ref: 5–49)												
50–200; > 200	1.26 (0.94–1.69)		1.22 (0.94–1.60)		1.66 (1.26–2.21)⁎		1.04 (0.78–1.39)		1.70 (1.24–2.37)⁎		1.28 (0.98–1.68)	
Work-related PA (ref: inactive)												
Active	0.31 (0.16–0.57)⁎⁎		0.10 (0.05–0.20)⁎⁎		0.35 (0.20–0.62)⁎⁎		0.48 (0.27–0.85)⁎		0.66 (0.26–1.55)		0.16 (0.09–0.30)⁎⁎	

*Worksite supports and policies*												
Schedule flexibility (ref: no)												
Yes	1.21 (0.94–1.56)		0.80 (0.62–1.02)		1.76 (1.40–2.23)⁎⁎		0.86 (0.69–1.07)		1.27 (1.02–1.59)⁎		1.01 (0.80–1.26)	
PA break (ref: no)												
Yes	0.84 (0.32–2.17)		1.35 (0.54–3.48)		0.63 (0.26–1.49)		1.25 (0.58–2.69)		1.28 (0.61–2.71)		1.08 (0.47–2.51)	
Stair prompt signage (ref: no)												
Yes	0.79 (0.34–1.77)		1.23 (0.54–2.92)		2.28 (1.07–5.00)⁎		2.66 (1.20–6.14)⁎		0.70 (0.32–1.49)		2.29 (1.02–5.29)⁎	
Workplace map (ref: no)												
Yes	1.28 (0.56–2.95)		0.67 (0.28–1.59)		1.01 (0.49–2.11)		0.56 (0.22–1.38)		0.80 (0.36–1.75)		1.19 (0.50–2.84)	

All models adjusted for age, sex, race and BMI.

⁎ *p* < 0.05.

⁎⁎ *p* < 0.001.

1 Occupational sitting time reference group is different in different occupation groups.
